# Diagnostic measures for severe acute malnutrition in Indian infants under 6 months of age: a secondary data analysis

**DOI:** 10.1186/s12887-021-02629-9

**Published:** 2021-04-01

**Authors:** Ranadip Chowdhury, Nitika, Tarun Shankar Choudhary, Sunita Taneja, Jose Martines, Nita Bhandari, Rajiv Bahl

**Affiliations:** 1 Knowledge Integration and Translational Platform (KnIT) at Centre for Health Research and Development, Society for Applied Studies, 45, Kalu Sarai, New Delhi, 110016 India; 2 Centre for Intervention Science in Maternal and Child Health, Centre for International Health, University of Bergen, Bergen, Norway; 3 Department of Maternal, Newborn, Child and Adolescent Health and Ageing, World Health Organization, Geneva, Switzerland

**Keywords:** Severe acute malnutrition, Weight for length z-score, Weight for age z-score, Length for age z-score, Under 6-month infants, Mortality

## Abstract

**Background:**

Weight for length z-score (WLZ) < − 3 is currently used to define severe acute malnutrition (SAM) among infants. However, this approach has important limitations for infants younger than 6 months of age as WLZ cannot be calculated using WHO growth standards if infant length is < 45 cm. Moreover, length for age z-score (LAZ) and weight for length z-score (WLZ) are least reliable measures, with high chances of variation, and less chances of detecting undernutrition in under 6 months infants. The objective of the current analysis was to compare WLZ with WAZ and LAZ in a cohort of Indian infants in predicting the deaths between 6 weeks and 6 months of age.

**Methods:**

The data was from an individually randomized trial conducted in slums of Delhi, India in which infants’ weight and length were measured at 6 weeks of age (at the time of the first immunization visit). Vital status of the infants was documented from 6 weeks to 6 months of age. The sensitivity, specificity, positive and negative predictive values, and positive and negative likelihood ratios were calculated for WAZ < -3, WLZ < -3, and LAZ < -3 for deaths between 6 weeks and 6 months of age. The receiver operating characteristics curve was calculated for each of the above anthropometric indicators.

**Results:**

For deaths occurring between 6 weeks to 6 months of age, the specificity ranged between 85.9–95.9% for all three anthropometric indicators. However, the sensitivity was considerably higher for WAZ; it was 64.6% for WAZ < -3, 39.1% for LAZ < -3, and 25.0% for WLZ < -3. WAZ < -3 had higher area under curve (0.75; 95% CI: 0.68, 0.82) and hence, better discriminated deaths between 6 weeks and 6 months of age than WLZ < -3. The adjusted relative risk (RR 10.6, 95% CI 5.9, 18.9) and the population attributable fraction (PAF 57.9, 95% CI 38.8, 71.0%) of mortality was highest for WAZ < -3.

**Conclusions:**

We found WAZ < -3 at 6 weeks of age to be a better predictor of death in the 6 weeks to 6 months of life in comparison to WLZ < -3 and LAZ < -3 and propose that it should be considered to diagnose SAM in this age group.

## Background

The first 6 months of life are marked by rapid growth, and neurological development [1]. During this period, optimal nutrition is through exclusive breastfeeding [2]. However, studies have identified high rates of non-exclusively breast-fed infants in this age group. Suboptimal practices predispose infants to undernutrition or growth faltering [3–6]. In low- and middle-income countries, 3.8 million under 6 months infants are severely wasted by weight for length z-score (WLZ < − 3) [7]. Also, in India, the burden of severe wasting is quite high (14.8%) among infants less than 6 months of age, as reported in the National family Health Survey-4 (NFHS-4, 2015–16) [8]. Based on alarmingly high rates of severe wasting in this age group, WHO updated its severe acute malnutrition (SAM) guidelines including for under 6 months infants for the first time in 2013 [9].

Diagnosis and management of SAM in under 6 months infants deserves special attention to reduce the global burden of SAM in under-5 children [9]. For children in the age group 6–59 months, WLZ < -3 is one of the criteria used to identify SAM, and this criterion was set based on the high risk of mortality observed in children below in comparison to children above this cut-off [10]. Due to lack of high-quality evidence, the same definition i.e., WLZ < -3 was suggested for infants under 6 months of age [9]. The use of WLZ criteria in infants less than 6 months has some limitations. Firstly, the WLZ cannot be calculated using the WHO 2006 growth standards if length of an infant is less than 45 cm, WLZ cannot be measured in a proportion of preterm or small for gestational age infants [11, 12]. Secondly, an accurate measurement of length is difficult in community settings in small infants as they tend to be in knee-bent position [13, 14]. Thirdly, it has been shown in African settings, that anthropometric parameters using length i.e., length for age z-score (LAZ) and weight for length z-score (WLZ) are least reliable measures, with high chances of variation, and less chances of detecting undernutrition in under 6 months infants [15, 16]. However, chances of measurement error are also high for hanging weighing scales due to lack of calibration, deviation from accurate measurement over time as the springs stretch out etc. [17]

Realizing the importance, the WHO and a recent Child Health and Nutrition Research Initiative prioritized establishing diagnostic criteria for SAM among under 6 months infants as one of the priority research questions [10, 18]. Limited evidence on the association between anthropometric indicators and mortality among under 6 months infants is available from South Asia where the burden of SAM is higher compared to other parts of the world [11, 12].

We compared WLZ with WAZ and LAZ when measured at 6 weeks of age (at the time of first immunization) in predicting deaths between 6 weeks and 6 months of age, using data from an individually randomized trial conducted in the urban slums of Delhi, India.

## Methods

### Study description

The is a secondary data analysis from an individually randomized, double blinded placebo-controlled trial conducted during 1995–97 to assess the safety and benefits of maternal postpartum, and infant vitamin A supplementation administered with each of the three diphtheria-tetanus-pertussis (DPT) and poliomyelitis immunizations and with a fourth dose with measles immunization on vitamin A status, anthropometric indicators, and severe morbidity during infancy [19]. This was a multi-country trial conducted in, India, Ghana and Peru. In this analysis we have presented findings from the Indian cohort where 4000 mother-infant dyads were enrolled 18–28 days after childbirth, from two slums of Delhi, Dakshinpuri and Tigri. The details of the original study are described elsewhere [19].

### Anthropometry measurements

The weights and lengths of infants were measured at the time of the first immunization scheduled at 6 weeks of age. Weights were measured using hanging spring scales accurate to 100 g, calibrated daily [19]. Lengths were measured using rigid length boards with a sliding foot scale accurate to 1 mm [19]. The team was trained using standard operating procedures for all measurements. Retraining was done based on the feedback received through monitoring by supervisors. Inter and intra observer standardization exercises were done before initiation of the study and periodically during the study [20]. Length was measured in triplicate, and the median was used, while the weight was measured once. Information regarding vital status of the infants was collected through household visits, every 4 weeks.

For this analysis, an infant was included if the weight and length was measured within 2 weeks of the first scheduled immunization at 6 weeks of age.

### Statistical methods

All the analyses were done using Stata 15.0 (Stata Corp, College Station, TX, USA). WAZ, WLZ, and LAZ scores were calculated using WHO standards [21] Infants with extreme anthropometric z-score values (LAZ, and WAZ values < − 6 and > + 6, and WLZ values < − 5 and > + 5) were excluded from the analysis [22]. The sensitivity, specificity, positive and negative predictive values, and positive and negative likelihood ratios were calculated for WAZ < -3, WLZ < -3, and LAZ < -3 for deaths between 6 weeks and 6 months of age. The sensitivity was defined as the proportion of infants having z-scores <− 3 at 6 weeks of age among those who died between 6 weeks to 6 months of age. Specificity was defined as the proportion of infants having z-scores ≥ − 3 at 6 weeks of age among those who survived between 6 weeks to 6 months of age. Positive predictive value (PPV) was defined as the proportion of infants who died between 6 weeks to 6 months of age among those had z scores <− 3 at 6 weeks of age. Negative predictive value (NPV) was defined as proportion of infants who survived between 6 weeks to 6 months of age among those had z scores ≥ − 3 at 6 weeks of age. The positive likelihood ratio was calculated as sensitivity/ (1-specificity), and negative likelihood ratio as (1-sensitivity)/specificity.

The receiver operating characteristics ROC curve, an index of the test’s ability to discriminate between true positives and true negatives was calculated for each of the above anthropometric indicators. “DIAGT” command in Stata 15.0 was used to estimate sensitivity, specificity, predictive values, likelihood ratios, and area under curve (AUC) of anthropometric indicators for deaths between 6 weeks and 6 months of age [23]. The AUC for WAZ, WLZ, and LAZ were compared using “roccomp” command in STATA [24]. Also, the cut-off of anthropometric indicators with maximum AUC for death between 6 weeks and 6 months was computed using “cutpt” command in STATA [25].

The generalized linear model (GLM) of the binomial family with log link function was used to estimate the relative risk (RR) for mortality between 6 weeks and 6 months of age, for each anthropometric indicator. The models were adjusted for the intervention groups and sex of the infants. The population-attributable fraction for each anthropometric indicator was calculated using “punaf” command in Stata [26].

## Results

**Fig. 1:**
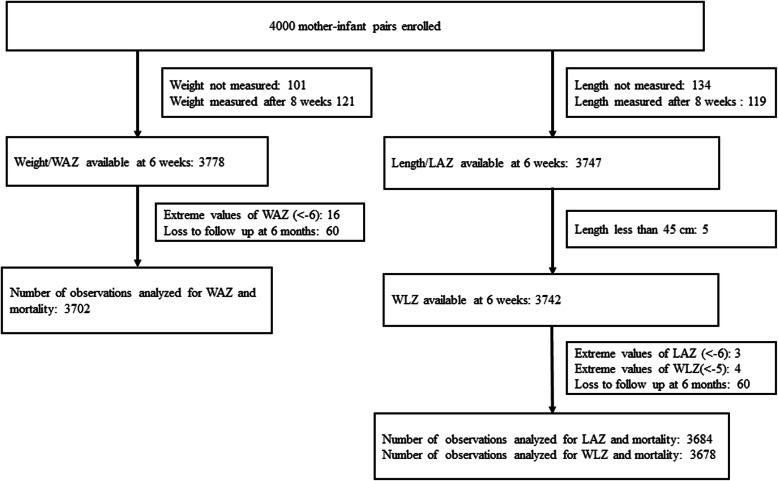
Flow chart showing the number of infants included in the analysis for different anthropometric indicators and reasons for exclusion. WAZ, Weight for age z-score; WLZ, Weight for length z-score; LAZ, Length for age z-score

The current analysis was done on 3702 infants for WAZ, 3678 infants for WLZ, and 3684 infants for LAZ (Fig. 1). The mean (standard deviation; SD) age of infants at the time of anthropometric assessment was 42.6 (1.6) days. The prevalence of WAZ < -3, WLZ < -3, and LAZ < -3 at the time of the first immunization were 14.7, 4.3, and 9.8% respectively (Table 1).

**Table 1:** Summary measures of anthropometric indicators

	WAZ (***n*** = 3702)	WLZ (***n*** = 3678)	LAZ (***n*** = 3684)
Mean (SD)	− 1.83 (1.16)	− 0.86 (1.15)	− 1.41 (1.18)
Prevalence of z-scores <−3 at 6 weeks (95% CI)	14.7 (13.5, 15.9)	4.3 (3.7, 5.0)	9.8 (8.9, 10.8)

*WAZ* Weight for age z-score, *WLZ* Weight for length z-score, *LAZ* Length for age z-score.

**Fig. 2:**
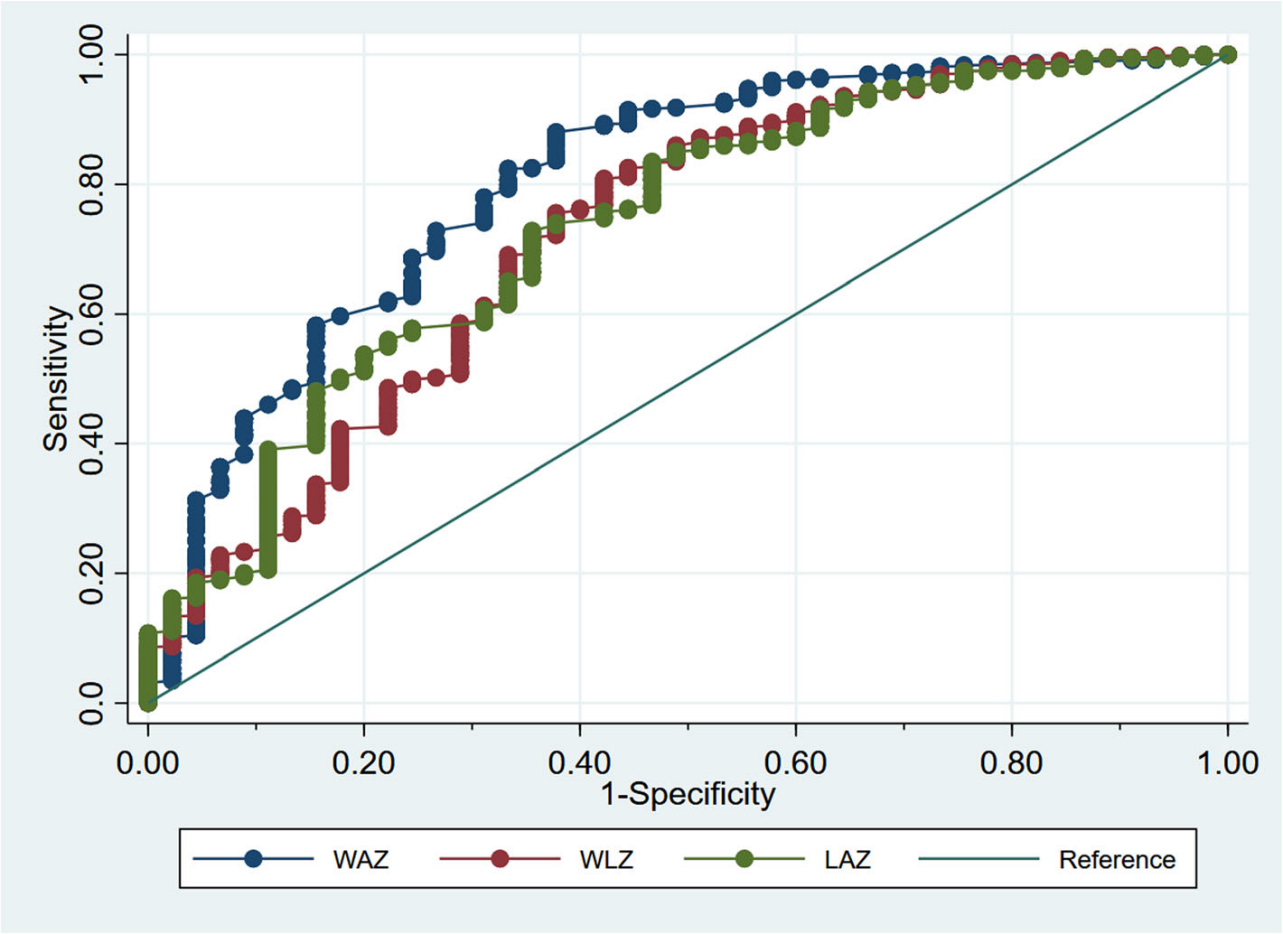
Receiver operating characteristics curve for WAZ, WLZ, and LAZ and deaths between 6 weeks and 6 months. WAZ, Weight for age z-score; WLZ, Weight for length z-score; LAZ, Length for age z-score

Figure 2 represents the ROC for WAZ, WLZ, and LAZ. The cut-offs with maximum AUC were − 3.15, − 1.78, − 1.64 for WAZ (AUC: 0.76), WLZ (AUC: 0.69), and LAZ (AUC:0.69), respectively. AUC was significantly different (*p* value < 0.001) between three anthropometric indicators.

Table 2 presents the diagnostic accuracy measures at “-3” cut-off for WAZ, WLZ, and LAZ. The sensitivity for deaths between 6 weeks and 6 months of age for WAZ < -3 was 64.6%, for WLZ < -3 was 25.0%, and for LAZ < -3 was 39.1%. The specificity ranged between 85 and 95% for all three indicators.

**Table 2:** Nutritional status at 6 weeks as a predictor of death between 6 weeks and 6 months of age

Nutritional Status at 6 weeks	Sensitivity (95% CI)	Specificity (95% CI)	Positive predictive value (95% CI)	Negative predictive value (95% CI)	Likelihood Ratio+ (95% CI)	Likelihood Ratio- (95% CI)
WAZ < -3	64.6 (49.5, 77.8)	85.9 (84.8, 87.0)	5.7 (3.9, 7.9)	99.5 (99.1, 99.7)	4.6 (3.7, 5.8)	0.4 (0.3, 0.6)
WLZ < -3	25.0 (13.2, 40.3)	95.9 (95.2, 96.5)	6.9 (3.5, 12.0)	99.1 (98.7, 99.4)	6.1 (3.6, 10.5)	0.8 (0.6, 0.9)
LAZ < -3	39.1 (25.1, 54.6)	90.6 (89.6, 91.5)	5.0 (2.9, 7.8)	99.2 (98.8, 99.4)	4.2 (2.9, 6.1)	0.7 (0.5, 0.8)

*WAZ* Weight for age z-score, *WLZ* Weight for length z-score, *LAZ* Length for age z-score.

WAZ < -3 had higher AUC (0.75; 95% confidence interval (CI): 0.68, 0.82) compared to WLZ < -3 (0.60; 95% CI: 0.54, 0.67) and LAZ < -3 (0.65; 95% CI: 0.58, 0.72).

Table 3 presents the risk of death between 6 weeks and 6 months of age. The overall mortality rate from 6 weeks to 6 months of age was 1.3% (48 out of 3702). The mortality rate in WAZ < -3, WLZ < -3 and LAZ < -3 groups were 5.7, 6.9 and 5%, respectively. The adjusted relative risk for mortality was highest for WAZ < -3 (10.6, 95% CI: 5.9, 18.9). The population-attributable fraction for mortality was highest for infants with WAZ < -3 (57.9, 95% CI: 38.8, 71.0%).

**Table 3:** Nutritional status at 6 weeks and relative risk and population attributable fraction of mortality between 6 weeks and 6 months of age

Anthropometric indicators at 6 weeks of age	Deaths among malnourished (%)	Deaths among non-malnourished (%)	Unadjusted Relative Risk (95% CI)	Adjusted Relative Risk^a^ (95% CI)	Population Attributable fraction % (95% CI)
WAZ	31 of 545 (5.7)	17 of 3157 (0.5)	10.6 (5.9, 18.9)	10.6 (5.9, 18.9)	57.9 (38.8, 71.0)
WLZ	11 of 159 (6.9)	33 of 3519 (0.9)	7.4 (3.8, 14.3)	7.3 (3.8, 14.2)	21.6 (7.5, 33.6)
LAZ	18 of 360 (5)	28 of 3324 (0.8)	5.9 (3.3, 10.6)	6.0 (3.4, 10.8)	32.5 (15.2, 46.3)

^a^Adjusted for intervention group and sex

*WAZ* Weight for age z-score, *WLZ* Weight for length z-score, *LAZ* Length for age z-score

Malnourished, WAZ < -3 or WLZ < -3 or LAZ < -3; Non-malnourished WAZ ≥ − 3 or WLZ ≥ -3 or LAZ ≥ -3.

## Discussion

Our analysis showed that WAZ < -3 has higher diagnostic accuracy to predict deaths between 6 weeks and 6 months in comparison to WLZ < -3 or LAZ < -3.

Similar to our findings, a community-based study conducted in Burkina Faso showed that infants with WAZ < -3 at 2 months of age had increased risk of mortality during infancy; this was not observed with WLZ < -3 [11]. A study conducted in Kenya showed that WAZ measured in infants between 1 and 6 months of age was one of strongest predictors of inpatient and post-discharge mortality during infancy [12]. However, the study was done among hospitalized infants who had higher risk of mortality compared to community cohorts of healthy infants. Due to the higher sensitivity and AUC, use of WAZ < -3 in comparison to WLZ < -3, will detect larger proportions of infants at risk of death and have better discriminatory ability to distinguish between infants at risk of dying from those not at risk. For the community-based detection of SAM, WAZ is relatively easy and more efficient as it requires measuring only weights; these are usually measured during home visits and in immunization clinics and recorded on growth cards used in national programs. Considering the better diagnostic accuracy, and ease of use in community settings, we propose that WAZ < -3 be considered for screening as well as diagnosis of SAM among under 6 months infants at the community and facility level.

It may be challenging to have different anthropometric screening criteria for infants younger than 6 months and for older infants and children. However, using WAZ for screening simplifies the assessment by relying on a single measurement (weight) and avoids depending on the measurement of lengths that is much harder to obtain in young infants. This challenge could be overcome through training of front-line health workers and medical officers [15]. Also, the use of WAZ criteria will lead to identification of increased number of SAM cases, as in this analysis we found that 14.7 and 4.3% infants were identified as SAM using the criteria of WAZ < -3 and WLZ < -3, respectively. And, if we translate this to around 27 million birth in a year in India, 3.90 million (14.7%) will be identified as SAM using the criteria of WAZ < -3 and 1.16 million (4.3%) using WLZ < -3. Thus, the use of WAZ < -3 will lead to 2.7 million additional cases of SAM annually compared to if WLZ < -3 is used. This would require strengthening the health system in terms of inpatient and outpatient services for infants identified as SAM, as infants with any complications such as any serious medical or clinical condition, recent weight loss or failure to gain weight, ineffective feeding, pitting edema, and any medical or social issue would need a more detailed assessment, and even hospital admission [9].

Ideally, in a program setting, one would like to identify as many as possible infants who are at higher risk of death in the next few months, as a result of severe malnutrition. For this, using WAZ as a tool in households and community surveys will be appropriate. Length measurement at the treating facility could be a part of the further assessment of these severely malnourished infants.

The strengths of this analysis are the large numbers, weight and lengths measured by a standardized team and minimal loss to follow up (1.5%). Some limitations are firstly, data used for analysis is around 20 years old. However, the relationship between anthropometric measures at 6 weeks and deaths between 6 weeks and 6 months observed, will be valid even today [12]. Secondly, we assessed diagnostic accuracy of anthropometric indicators assessed at 6 weeks of age, so we cannot comment on how anthropometric measures were associated with mortality in the first 6 weeks of life. Thirdly, we did not have information on weight and gestational age at birth.

## Conclusions

We found that WAZ < − 3 is a better predictor of mortality between the ages of 6 weeks and 6 months compared to WLZ < -3 and LAZ < -3. These results support the consideration of using WAZ for screening and diagnosis of SAM in the first 6 months of life, as it will aid in identifying children at risk of mortality in the Indian subcontinent and similar settings. These findings should be confirmed through well-designed prospective studies for addressing the definition of SAM in infants < 6 months of age in low- and middle-income countries (LMICs). Further, evaluation of MUAC for its role in screening of SAM in the first 6 months of life in Indian settings would allow policymakers to base decisions on all potential options.

## Data Availability

The data pertaining to the current analysis may be sent to the corresponding author, Ranadip Chowdhury (ranadip.chowdhury@sas.org.in).

## Abbreviations

AUC: Area under curve
CI: Confidence interval
DPT: Diphtheria-Tetanus-Pertussis
GLM: Generalized linear model
LAZ: Length for age z-score
LMICs: Low- and middle-income countries- LMICs
NFHS-4: National Family Health Survey-4
NPV: Negative predictive value
PAF: Population attributable fraction
PPV: Positive predictive value
ROC: Receiver operating characteristics
RR: Relative risk
SAM: Severe acute malnutrition
SD: Standard deviation
WAZ: Weight for age z-score
WLZ: Weight for length z-score
